# Targeting KRAS in pancreatic cancer

**DOI:** 10.32604/or.2024.045356

**Published:** 2024-04-23

**Authors:** SANDRA STICKLER, BARBARA RATH, GERHARD HAMILTON

**Affiliations:** Institute of Pharmacology, Medical University of Vienna, Vienna, A-1090, Austria

**Keywords:** Pancreatic cancer, PDAC, KRAS, SOS1, PROTAC

## Abstract

Pancreatic cancer has a dismal prognosis due to late detection and lack of efficient therapies. The Kirsten rat sarcoma virus (KRAS) oncogene is mutated in up to 90% of all pancreatic ductal adenocarcinomas (PDACs) and constitutes an attractive target for therapy. However, the most common KRAS mutations in PDAC are G12D (44%), G12V (34%) and G12R (20%) that are not amenable to treatment by KRAS G12C-directed cysteine-reactive KRAS inhibitors such as Sotorasib and Adagrasib that exhibit clinical efficacy in lung cancer. KRAS G12C mutant pancreatic cancer has been treated with Sotorasib but this mutation is detected only in 2%–3% of PDAC. Recently, the KRAS G12D-directed MRTX1133 inhibitor has entered clinical trials and more of such inhibitors are in development. The other KRAS mutations may be targeted indirectly via inhibition of the cognate guanosine exchange factor (GEF) Son of Sevenless 1 that drives KRAS. These agents seem to provide the means to target the most frequent KRAS mutations in PDAC and to improve patient outcomes.

## Introduction

Pancreatic cancer is distinguished by one of the highest mortality rates among all malignant diseases [1,2]. Minor improvements in surgery and chemotherapy have resulted in a 5-year survival rate as low as 9% [3–5]. Approximately 90% of pancreatic cancers present as pancreatic ductal adenocarcinomas (PDAC) and up to 80% of these patients present with advanced disease and have no efficient treatment available [6]. The fraction of cases approximately 20% eligible for surgical care have a 5-year survival rate below 30% [7]. The dismal prognosis of pancreatic cancer is associated with the absence of early-stage-specific symptoms, the lack of valid screening tests, and the failure of current therapies to prolong survival [8]. Chemotherapy is applied for patients with advanced disease but is rendered ineffective due to acquired resistance. The first-line chemotherapeutic, Gemcitabine, for pancreatic cancer patients has been replaced by modified FOLFIRINOX (mFOLFIRINOX) (5-fluorouracil, leucovorin, oxaliplatin and irinotecan) or nab-paclitaxel plus gemcitabine therapies but these regimens provide only modest survival in presence of high toxicity [9,10]. Novel targeted therapies so far have failed to result in superior overall survival (OS) [11]. Still, in the case of highly active novel agents, the typical inflammatory fibrotic reaction (desmoplasia) that accompanies pancreatic cancer impairs drug delivery and promotes immune escape [12]. The stroma-rich tumor microenvironment (TME) that constitutes up to 90 percent of the tumor volume is characterized by a high interstitial fluid pressure and collapsing vascular supply [13,14]. Accordingly, cancer cells are deprived of blood-derived nutrients and oxygen. In light of the rising incidence of pancreatic cancer, new targets and treatment modalities are urgently needed [15]. Mutant Kirsten rat sarcoma virus (KRAS) is expressed in most cases of PDAC and strategies involving specific inhibitors that were found to work successfully in Non-Small Cell Lung Cancer (NSCLC) therapy may be adapted suitably for PDAC. For example, the KRAS G12C inhibitor Sotorasib has been modified to target KRAS G12D by removing the cysteine-reactive group and addition of residues to block this KRAS mutant and to fill the switch region binding pocket.

### Genetics of PDAC

**Figure 1:**
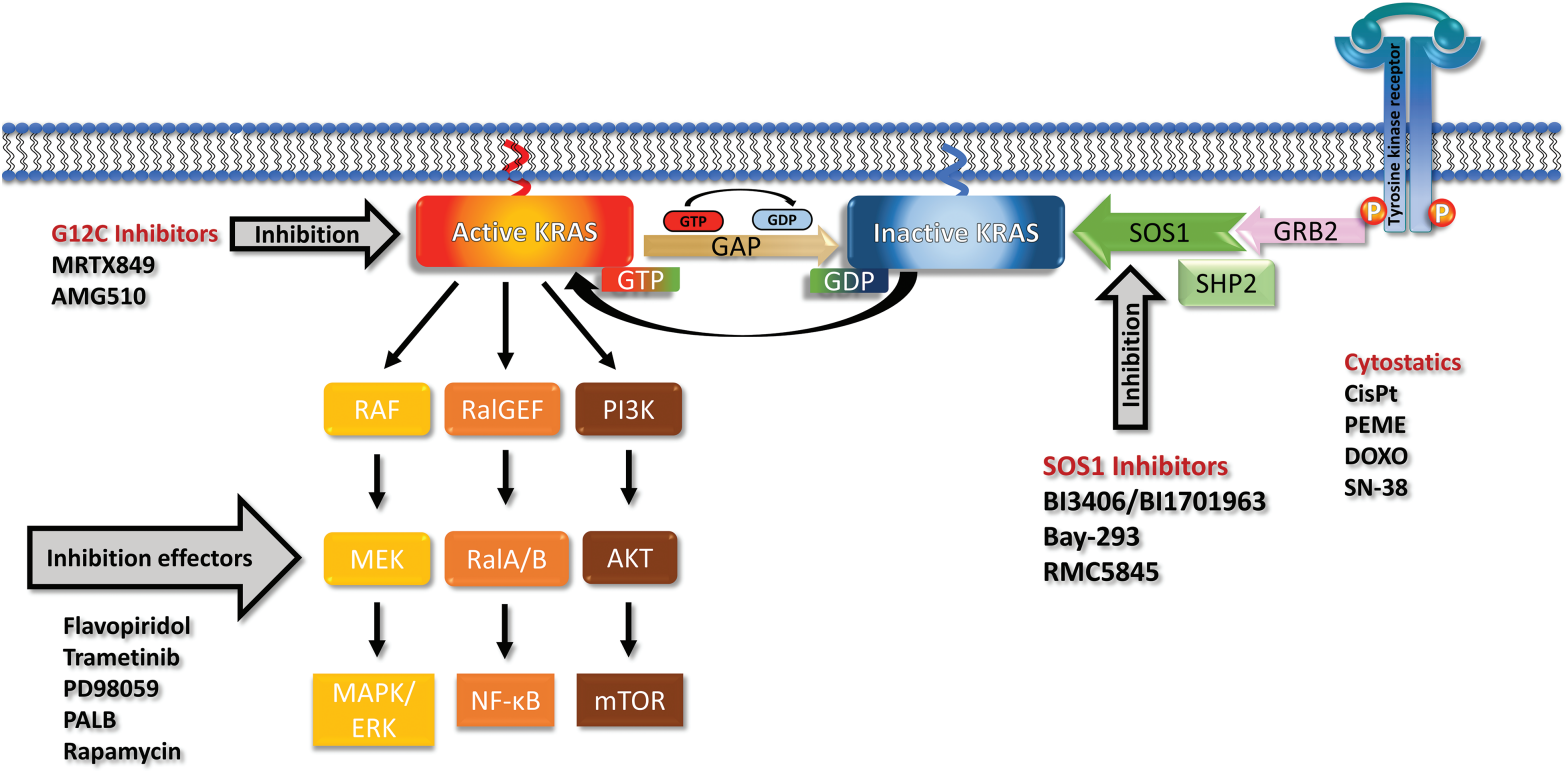
Receptor tyrosine kinases (RTK) recruit GRB2, SHP2 and SOS1 and load inactive KRAS (off state) with GTP to reach the active KRAS (on state). KRAS recycles through the action of Guanosine triphosphatase activating protein (GAP) back to the GDP-loaded inactive form. Mutations prevent the activity of GAPs and leave KRAS in a permanent overactive state that stimulates the downstream signaling pathways via the MAPK, PI3K and RAL cascades. The KRAS G12C can be blocked by Sotorasib or Adagrasib but no other mutated KRAS variants. The effects of mutated KRAS can be inhibited by inhibitors of the downstream signaling or cyclin-dependent kinase (CDK) inhibitors. SOS1 can be inhibited by a range of compounds including the BI-1701963, that is in clinical trials, and a range of other drugs. KRAS and SOS1 inhibitors may be combined with cytostatics to achieve more pronounced responses.

Genetically, PDAC is characterized by several frequent mutations in oncogenes and suppressor genes that cause progressive disease [16]. The four canonical mutations, namely KRAS, present in approximately 85%, TP53, CDKN2A, and SMAD4, in over 50% of cases, are typically present. Mutated KRAS is the initiating event in the early development of pancreatic neoplasia [17]. Although KRAS mutations are detectable in a high proportion of PDACs, almost in half of colorectal adenocarcinomas (CRC) and in approximately 30% of lung adenocarcinomas, higher response rates for KRAS-targeted therapy are so far restricted to lung cancer [18]. Mutations in the KRAS gene are concentrated in the three residues G12, G13, and Q61 impairing GTPase activity and locking KRAS in its active state [19,20]. GDP-GTP exchange by KRAS is promoted by the guanine nucleotide exchange factors (GEF), in particular by Son of Sevenless 1 (SOS 1), and GTPase-activating proteins (GAPs) that recycle KRAS to its inactive state (Fig. 1) [21]. The KRAS downstream signaling pathways include the MAP-kinases (MAPK), the PI3-kinase (PI3K)/AKT/mTOR pathway, and the small GTPases Rho, Rac, and Ral driving proliferation, survival, metabolic adaptions, and tumor growth. Besides tumor growth, the excessive activation of KRAS in PDAC regulates cellular activities such as metabolism, autophagy, macropinocytosis, and TME [22–25].

### Targeting of mutated KRAS in PDAC

**Figure 2:**
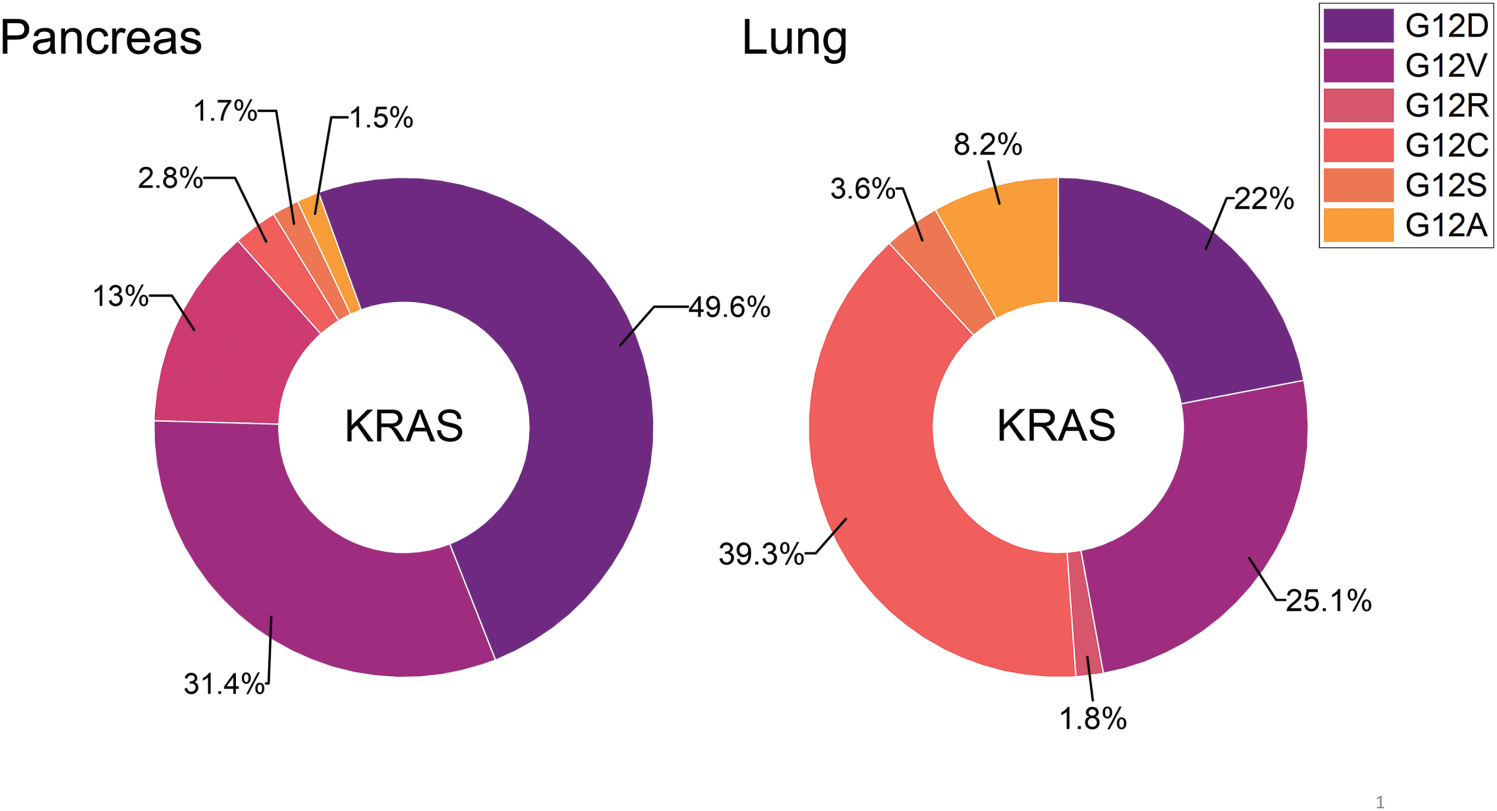
The distribution of distinct KRAS mutations is compared for pancreatic and lung cancer. So far, KRAS G12C is amenable to treatment with the approved drugs Sotorasib or Adagrasib but this mutant KRAS allele is present in a very low percentage in pancreatic cancer.

The now targetable KRAS G12C is detectable only in 1%–3% of PDACs [22,23]. In detail, incidences of the other KRAS mutations in PDAC include G12D (44%), G12V (34%), G12R (20%), and Q61H (4%) (Fig. 2) [17]. In general, studies have shown that KRAS mutations could be considered a marker for a poor prognosis for pancreatic cancer [26,27]. KRAS has been considered undruggable for a long time [28,29]. Recently, the KRAS inhibitors Sotorasib (AMG 510/Lumakras) and Adagrasib (MRTX849/Krazati) directed to the Cys12 amino acid residue of the active center have achieved significant clinical responses in NSCLC [30,31]. However, KRAS-mutated cancers carrying non-G12C mutations are not amenable to such an approach due to the lack of a reactive cysteine in its active site [32].

### Clinical treatment of KRAS G12C in pancreatic cancer

Sotorasib has been tested in the multicenter, phase 1/2 CodeBreak 100 clinical trial to assess the safety and efficacy in KRAS G12C-mutated solid cancers after at least one prior therapy line (NCT03600883). First data demonstrated significant activity in PDAC, with 6/8 patients exhibiting stable disease and three showing a 30% reduction at 4.3 months median follow-up [33]. An updated report of 38 patients with pretreated PDAC (mean age: 65 years, 76.3% male, median 2 lines of previous chemotherapy) from the combined phase I/II study receiving Sotorasib 960 mg once daily has been published [34]. Eight patients had a partial response resulting in a response rate of 21% and a disease control rate of 84.2%. The median progression-free survival was 4.0 months, and the median overall survival was 6.9 months, respectively. Overall, treatment-related adverse events were reported in 16 patients (42%) with 6 patients (16%) experiencing grade 3 events. Thus, Sotorasib demonstrated clinical anticancer activity and tolerability. However, in NSCLC the results of Sotorasib are superior with an overall response rate of 41%, a progression-free time of 6.3 months, and an overall survival time of 12.5 months [35].

Adagrasib has been applied in the KRYSTAL-1 trial for a cohort of ten patients with pretreated KRAS G12C-mutant PDAC [36]. All participating patients revealed clinical benefit, including 5/10 (50%) partial responses and a progression-free survival of 6.6 months. In comparison, a response rate of approximately 10% and a median progression-free survival of less than 3 months is typically found in second-line chemotherapy for PDAC [37]. An update reported responses in 7/21 (33.3%) of the patients with PDAC and 5/12 (41.7%) responses in biliary tract cancers [38]. These results need to be tested in larger trials, alone or in combination with other agents. However, as proof of principle, these KRAS-targeting drugs were effective in reaching the pancreatic tumor tissue despite the manifold adverse effects of the TME. Overall, the response rates and durations of efficacy are disappointing compared to the positive treatments observed in NSCLC.

### KRAS G12D inhibitors

#### MRTX1133

About 93% of PDACs have KRAS mutations, with G12D and G12V being the most common genetic alterations [39]. It proved difficult to develop inhibitors of the other KRAS alleles, such as G12D which is most common in pancreatic cancer. KRAS G12D lacks the reactive cysteine residue near the switch II region making the covalent blockade of the KRAS active center impossible in this allele. Based on the structure of Adagrasib, optimization steps involving unusual hydrogen bonding and ion pair binding for the Asp12 residue of KRAS G12D led to the development of MRTX1133 (Mirati Therapeutics, San Diego, CA, USA). This G12D inhibitor binds to GDP-bound KRAS G12D with nanomolar affinity and over 700-fold higher affinity compared to wild-type KRAS [40]. MRTX1133 exhibits single-digit nanomolar activity in cellular tests, suppresses KRAS G12D signaling and it showed high antitumor activity in murine animal models [40]. In detail, this inhibitor showed tumor regression >30% in 73% (8/11) of KRAS G12D-mutated PDAC cell lines and patient-derived xenografts (PDX) [40]. MRTX1133 is currently in first clinical studies labeled “MRTX1133 in Patients With Advanced Solid Tumors Harboring a KRAS G12D Mutation” (NCT05737706) [41,42]. This is a Phase 1/2 multicenter study checking the safety and antitumor activity of MRTX1133 in patients with KRAS G12D mutant solid tumors.

For MRTX1133 and KRAS G12D pancreatic cancer cell lines, IC_50_ values of 0.42 nM and 4.8 nM for ASPC-1 and 3.6 nM for HPAF-11 have been reported [41]. These values have been determined in three-dimensional (3D) cultures of the cell lines on Matrigel. MRTX1133 has much higher antiproliferative activity in 3D compared to two-dimensional (2D) cultures, that may lack chemosensitivity [43]. However, significant fractions of the cells up to 30% are still viable at concentrations of 4 µM of MRTX1133 [39]. In a study checking the cell lines in 2D cultures, IC_50_ sensitivities ranged from >100 to >5,000 nM for MRTX1133 (cell lines: LS513 > 100, HPAF-11 > 1,000, SNUC2B > 5,000, PANC-1 > 5,000) [44]. For CRC lines with G12V, G13D, or WT KRAS IC_50_ values for MRTX1133 ranged from 1 to over 5 µM. However, activity of MRTX1133 against other KRAS mutant alleles and wild-type KRAS proteins has been reported [45]. In combination with relatively poor bioavailability, survival of cells at higher concentrations of MRTX1133, and possible development of chemoresistance this drug may experience limitations in clinics. An oral prodrug of MRTX1133, prodrug 9, exhibited better bioavailability in mice and was active in a KRAS G12D mutant xenograft mouse tumor model [46]. A study employing isogenic cell lines that express a single KRAS allele demonstrated that MRTX1133 also exhibited significant activity against KRAS mutations including G12C, G12V, G13D, and wildtype KRAS but spare HRAS and NRAS because the binding to H95 of KRAS, a residue that is not conserved in HRAS and NRAS, is essential for MRTX1133 activity [45].

### Resistance to MRTX1133

For all mutant KRAS, inhibitor treatments intrinsic and acquired chemoresistance has been observed [39]. Some KRAS G12D mutated PDAC cell lines are not sensitive to MRTX1133 treatment [43]. Due to the high-affinity binding of MRTX1133 to mutant KRAS additional point mutations near the switch-II pocket or reactivating of alternative signaling cascades may confer resistance [47]. In addition to the driver KRAS mutations cells exhibit inactivation of tumor suppressors including CDKN2A, TP53, SMAD4 and PTEN as well as activation of PIK3CA that promote the progression and resistance of PDACs [39]. The development of effective MRTX1133 combination therapies, with signal transduction inhibitors or immunotherapy, are expected to increase the antitumor efficacy compared to use as a single agent.

MRTX1133 shows low affinity against wild-type KRAS protein but may reactivate upstream signaling events by upregulating the expression and phosphorylation of EGFR and HER2 [42]. Accordingly, the combination of MRTX1133 with the EGFR inhibitors Cetuximab or Afatinib have been reported to show increased anticancer activity in xenograft models [48,49]. The PIK3CA inhibitor Alpelisib was found to synergize with MRTX1133 but no synergy has been detected with MEK or ERK inhibitors. Cancer cells with acquired resistance to MRTX1133 *in vitro* remained sensitive to this Afatinib combination therapy [50]. Possible synergy with current PDAC first-line treatments such as FOLFIRINOX chemotherapy or other drugs need to be established.

### MRTX1133 and the tumor immune microenvironment (TIME)

PDAC is characterized by a low survival time of approximately 8 months but trials to improve this dismal prognosis by application of novel immunotherapies with checkpoint inhibitors have failed so far [51]. This negative result points to a desmoplastic and immunosuppressive tumor immune microenvironment that interferes with this approach. In detail, a dense tumor stroma containing cancer-associated fibroblasts (CAF), and the presence of suppressive immune effectors, such as cancer-associated macrophages (CAM) and myeloid-derived suppressor cells (MDSC) hinder infiltration and activity of antitumor immune cells [52].

In addition to killing tumor cells, MRTX1133 treatment induced marked changes in the TIME [39]. MRTX1133 treatment for one week leads to significant modifications of the PDAC TIME of immunocompetent mice [53]. These changes included readjustments of CAFs and remodeling of the tumor stroma. Furthermore, increased vascular density, acquisition of the M1 phenotype in macrophages, elevated exclusion of suppressive myeloid cells, and increased influx of CD8+ T cell lymphocytes were induced by MRTX1133 [43]. Such preclinical data suggest that the active immune system increases the antitumor effects of MRTX1133. Actually, immune-deficient mouse models lacked therapeutic efficacy of the combination of MRTX1133 and CTLA-4 and/or PD-1 checkpoint inhibitors [43,49,54].

### Other KRAS G12D inhibitors

KRAS G12C aside, more than 85% of KRAS-mutant cancers are still in need of effective treatment. Furthermore, response rates to Sotorasib and Adagrasib are only around 40%, so there’s room for improvement all around. Other KRAS G12D-directed small-molecule inhibitors are at an earlier phase of development, such as HRS-4642 (Jiangsu Hengrui Medicine, Lianyungang, China; phase I; NCT05533463), BI-KRASG12D (Boehringer Ingelheim, Germany), JAB-22000 (Jacobio, Beijing, China), ERAS-4 (Erasca) TH-Z835 and RMC-6236 among others [48].

However, HRS-4642 shows just one partial response among 13 evaluable patients in a phase 1 trial across various solid tumors in a subject receiving 200 mg [55]. The study is testing HRS-4642 at 15-300 mg per week and liver toxicity and grade 3 or greater hypercholesterolaemia was observed in 17% of HRS-4642-treated patients. The TH-Z835 inhibitor associates with the switch-II pocket and inhibits KRAS G12D by formation of a salt bridge between the piperazine moiety of the inhibitor and the Asp12 residue [56]. TH-Z835 has been demonstrated to suppress MAPK signaling and cell proliferation as well as growth of xenografts in models of pancreatic cancer and additionally shows synergy with an anti-PD-1 checkpoint antibody.

RMC-6236 represents a first-in-class oral KRAS (ON) inhibitor that exerts high antitumor activity in a range of KRAS-driven cancer models, including G12A/C/D/R/S/V and additionally H- and NRAS [57]. Oral administration of RMC-6236 in PDAC KRAS-mutant xenografts resulted in tumor regressions with low side effects [58]. To date, 65 patients with PDAC have been enrolled in the trial’s dose-escalation phase of RMC-6236, with tumors harboring variants other than KRAS G12C. G12D in never smokers made up 35% of this group. In the PDAC cohort, the Overall Response Rate (ORR) was 20% and the Disease Control Rate (DCR) was 87% among 46 evaluable patients. RMC-6236’s side effects included rash, nausea, and diarrhea. Pancreatic cancer patients with KRAS G12D, G12R, and G12V variants responded to treatment.

BI-2852 is a KRAS inhibitor that targets between switch I and II regions on KRAS G12D with nanomolar affinity [59]. It inhibits all interactions of GEFs and GAPs with KRAS, resulting in decreased downstream signaling and antiproliferative effects in KRAS mutant cells. The biological effects of this compound, at least in part, seem to be due to the formation of the KRAS dimers. Boehringer Ingelheim has reported the discovery of pan-KRAS inhibitors active against KRAS G12C-, G12D-, G12V- and G13D-driven cells. These pan-KRAS inhibitors and proteolysis targeting chimeras (PROTACs) spare NRAS and HRAS proteins [60].

### Targeting KRAS by SOS1 inhibitors

An approach to target all mutant KRAS alleles indiscriminately is the inhibition of upstream effectors that support the function of KRAS. Two promising targets are interference with KRAS nucleotide exchange via the GEF SOS1 or inhibition of SHP2 both involved in normal KRAS function (Fig. 1) [61]. SHP2 is a phosphatase with diverse cellular activities, including the activation of RAS by partially unclear mechanisms [62,63]. The oral small-molecule SOS1 inhibitor BI-3406 disrupts the SOS1–KRAS interaction for all KRAS alleles *in vitro* and in KRAS-dependent cancer models [64]. Furthermore, BI-3406 was shown to inhibit cellular proliferation in synergistic combination with MEK inhibitors [64]. The SOS1 inhibitor BI-1701963 is the first of such compounds that has reached clinical trials of KRAS-mutated advanced solid tumors alone or in combination with trametinib (NCT04111458). First data from a dose escalation trial employing BI-1701963 as monotherapy showed good tolerability and stable disease up to 18 weeks in 7/31 patients with KRAS mutated solid tumors [65].

The RM-0331 SOS1-directed compound (Revolution Medicines) targets the same binding site as BI-3406 [66,67]. A range of further SOS1 inhibitors is under development, including RMC-5845, Schrödinger SDGR5, Genhouse Bio GH52, Erasca ERAS-9, and MRTX0902 from Mirati Therapeutics [68,69].

### SOS1-directed PROTACs

PROTACs, a new class of drugs, specifically remove target proteins via the proteasomal degradation and exert much larger effects compared to simple inhibition of proteins by recycling to attack more antigens [70]. These bifunctional compounds couple the protein of interest and a E3 ligase, resulting in ubiquitination and subsequent proteolytic degradation [71]. An example of such a potent KRAS PROTAC is the BI-KRASdegrader1 that can direct all KRAS mutants to degradation while sparing NRAS and HRAS [60]. A SOS1-directed PROTAC (9d) was produced by linking a VHL ligand to the SOS1 agonist VUBI-1 that induced SOS1 degradation in various KRAS-mutated cancer cells with high antiproliferation activity [69,72]. Compound 9d exhibited high antitumor efficacy against human lung cancer xenografts. For the synthesis of SOS1 PROTACs only binding to SOS1 is required, independently of the function as inhibitor or agonist. At present it is not clear whether these PROTACs will be prone to acquired resistance, since the PROTAC machinery is subject to their own specific ways of resistance [71]. Such pan-RAS PROTACs may cause higher level of toxicity than KRAS allele-specific inhibitors. These pan-KRAS drugs would be suited for non-KRAS G12C tumors prominent PDAC and colon cancer.

## Discussion and Conclusion

Mutant KRAS variants constitute the prime target of directed therapies in PDAC due to their high frequent expression and function as an efficient driver of proliferation and survival [73]. Inhibition of KRAS specimens is a rather new achievement and has found effective clinical application in NSCLC and KRAS G12C-mutated tumors. Despite emerging resistance to KRAS-directed therapy, response rates of approximately 40% and high disease control rates could be achieved using Sotorasib and Adagrasib. Unfortunately, KRAS G12C is rare in PDAC and inhibitors directed to other KRAS mutations frequently found in this tumor type are in early clinical development. However, the rare KRAS G12C variant has been successfully treated in PDAC using the approved specific agents, despite the extensive and dense fibrous stroma that hinders drug delivery [74]. The first KRAS G12D, MRTX1133, exhibits high preclinical activity and modulates the TIME for better responsiveness to immunotherapy. A range of other G12D-directed inhibitors are under development. Aside from direct inhibition of mutant KRAS, targeting of SOS1 is a viable option as pan-KRAS therapy. The first SOS1 inhibitor, BI-1701963, is in clinical trials in different combinations and this drug as well as numerous followers will become available shortly to treat non-G12C KRAS variants as most frequently expressed in PDAC. Novel SOS1-directed PROTACs are expected to provide much higher efficacy in depleting SOS1 and blocking KRAS [75–78].

## Data Availability

Not applicable.
